# Natural Small Molecules Targeting Oxidative Stress and Redox Homeostasis in Aging: Mechanisms and Therapeutic Potential

**DOI:** 10.3390/antiox15050621

**Published:** 2026-05-14

**Authors:** Yu Li, Haodong Wu, Zeyi Zhang, Mingshan Wang, Yue Zhao, Huimin Sun

**Affiliations:** 1 School of Medicine, Xiamen University, Xiamen 361102, China; 2 Department of Urology, Xiang’an Hospital of Xiamen University, School of Medicine, Xiamen University, Xiamen 361102, China; 3 Central Laboratory, Xiang’an Hospital of Xiamen University, School of Medicine, Xiamen University, Xiamen 361102, China

**Keywords:** natural small molecules, ageing, age-related chronic inflammation, antioxidant activity, antioxidant activity, autophagy, mitochondrial function

## Abstract

The global population is ageing rapidly; adults aged ≥ 60 years are projected to exceed 2 billion by 2050. Ageing is a major risk factor for chronic and degenerative disorders and is increasingly viewed as a modifiable biological program. Oxidative stress is a central driver: sustained ROS/RNS accumulation damages lipids, proteins and nucleic acids and amplifies mitochondrial dysfunction, inflammaging, cellular senescence, impaired autophagy and telomere instability. Targeting these shared mechanisms may therefore deliver multi-disease benefits beyond single-disease therapy. Medicinal plants provide chemically defined monomers that can act as direct antioxidants and, more importantly, restore redox homeostasis by modulating conserved signaling axes, including Nrf2/FOXO/SIRT1, AMPK/mTOR and NF-κB. However, the current evidence base remains highly heterogeneous, and reliable clinical validation is still limited. In this review, we summarize studies published over the last decade on medicinal plant-derived monomers with reported anti-ageing relevance in the context of oxidative stress and redox homeostasis. We compare major redox-centered pathways, molecular targets, model systems, and outcome measures, and evaluate the evidence with attention to its strength, consistency, and translational relevance. Particular emphasis is placed on current limitations, including model dependence, variable bioavailability, uncertain dose–exposure relationships, and the lack of well-designed clinical studies. These considerations are intended to provide a more cautious and evidence-based framework for future mechanistic and translational research.

## 1. Introduction

Aging denotes the progressive decline in physiological function and diminished environmental adaptability occurring over time in organisms Ageing is not driven by a single factor, but rather results from the long-term combined effects of intrinsic and extrinsic environmental influences. The traditional “wear-and-tear” theory proposes that organisms are continuously subjected to mechanical, metabolic, and environmental damage throughout life, and as repair capacity gradually declines, the cumulative burden of such damage ultimately leads to functional deterioration of tissues and organs. In terms of intrinsic factors, genetic background, mitochondrial dysfunction, endocrine imbalance, immunosenescence, and reduced cellular repair capacity can all promote the accumulation of oxidative damage and the disruption of homeostasis. Among extrinsic factors, ultraviolet exposure, air pollution, smoking, unhealthy diet, sleep disturbance, and chronic psychological stress can further aggravate inflammatory responses and metabolic burden, thereby accelerating the ageing process [1,2,3].

Accumulating evidence has shown that aging is closely associated with cancer, cardiovascular diseases, neurodegenerative disorders, diabetes, and chronic inflammatory diseases, and constitutes an important risk background for the development and progression of multiple age-related chronic diseases [4]. Moreover, chronic diseases can further accelerate the aging process [5], creating a self-reinforcing vicious cycle that increases mortality [6,7]. Therefore, aging is not only a central topic in basic biological research, but also an important entry point for extending healthspan and reducing the burden of chronic disease.

**Figure 1:**
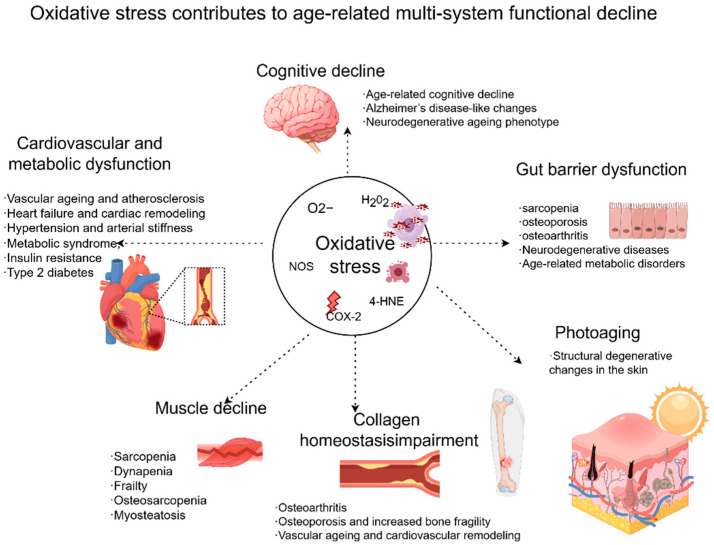
Oxidative stress as a central driver of age-related multi-system functional decline and disease-related phenotypes. (Oxidative stress acts as a central upstream driver of age-related dysfunction across multiple organs and tissues. Excessive reactive oxygen species (ROS), together with oxidative damage-related mediators and readouts such as H_2_O_2_, O_2_^−^, NOS, 4-HNE, and COX-2, are associated with functional decline and pathological changes in several biological systems. In the nervous system, oxidative stress contributes to cognitive decline, including age-related cognitive decline, Alzheimer’s disease-like changes, and neurodegenerative ageing phenotypes. In the cardiovascular and metabolic system, it is linked to vascular ageing and atherosclerosis, heart failure and cardiac remodeling, hypertension and arterial stiffness, metabolic syndrome, insulin resistance, and type 2 diabetes. In the gut, oxidative stress is associated with gut barrier dysfunction, which may contribute to sarcopenia, osteoporosis, osteoarthritis, neurodegenerative diseases, and age-related metabolic disorders. In skeletal muscle, oxidative stress promotes muscle decline, including sarcopenia, dynapenia, frailty, osteosarcopenia, and myosteatosis. In connective tissues, oxidative stress disrupts collagen homeostasis and is associated with osteoarthritis, osteoporosis and increased bone fragility, as well as vascular ageing and cardiovascular remodeling. In the skin, oxidative stress accelerates photoaging and contributes to structural degenerative changes).

Among the mechanisms involved in aging, oxidative stress and redox homeostasis imbalance are considered key nodes linking multiple aging-related phenotypes (Figure 1). Under physiological conditions, reactive oxygen species (ROS) participate in cell signaling, energy metabolism, and immune regulation. However, excessive ROS accumulation or impaired antioxidant defense can trigger a range of pathological events, including DNA damage, lipid peroxidation, oxidative protein modification, mitochondrial dysfunction, and chronic inflammatory activation. Although oxidative stress is not the sole determinant of aging, it is tightly associated with mitochondrial injury, proteostasis disruption, impaired autophagy, and cell fate regulation, and thus plays an important role in the aging regulatory network.

The advantages of medicinal plants in ageing intervention primarily lie in their multi-component, multi-targeted nature, integration of prevention and treatment, high safety profile, and potent synergistic effects. These attributes confer unique value to medicinal plants in anti-ageing research and applications. Research on individual natural small molecules targeting ageing has gradually increased, attracting widespread attention. Accordingly, this review focuses on natural small molecules derived from medicinal plants and summarizes recent advances in their anti-aging effects mediated by targeting oxidative stress and redox homeostasis.

To clarify the scope of inclusion in this review and enhance comparability across different studies, we systematically searched the PubMed database for relevant literature published between 2015 and 2025, and excluded studies on compound formulations, studies whose subjects did not fit the theme of this review, and non-English publications. On the basis of summarizing representative medicinal plant-derived natural monomers and their associated signaling pathways, this review further compares and synthesizes evidence across different experimental levels, and analyzes the major limitations of current research, with the aim of providing a more evidence-based reference framework for future mechanistic exploration and translational studies.

## 2. Overview of Natural Small Molecules

Single compounds derived from medicinal plants are isolated and purified from herbal sources, each exhibiting distinct chemical structures and notable biological activities. These compounds serve as essential entities for mechanistic investigation within complex herbal formulations. While medicinal plants achieve synergistic multi-component and multi-target effects through herbal formulae [8], modern pharmacological research increasingly emphasises the isolation, identification, and mechanistic elucidation of individual compounds. The study of natural small molecules has transitioned from empirical formulations to systematic scientific investigation. Historically, the discovery and application of representative monomers such as berberine, ephedrine, artemisinin, tanshinone IIA, and andrographolide established the foundation for the modernisation of medicinal plants and the development of new pharmaceuticals According to available statistics, approximately one-fifth of the global population uses medicinal plants, with the anti-malarial success of artemisinin representing a landmark achievement in modern pharmacology [9].

**Figure 2:**
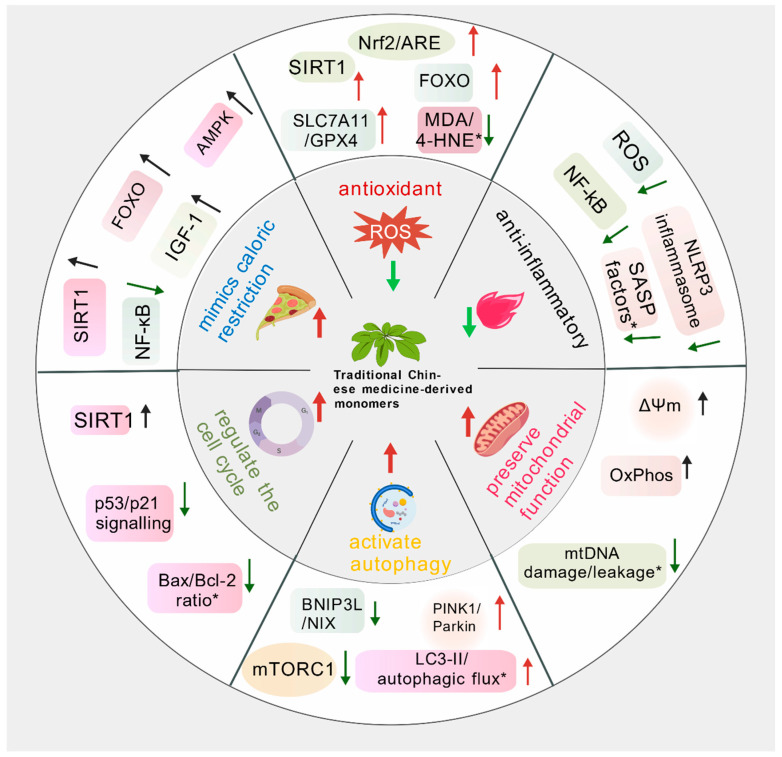
Representative anti-ageing molecular modules and functional readouts influenced by traditional Chinese medicine-derived monomers. (This schematic summarizes the major molecular modules through which traditional Chinese medicine-derived monomers may counteract ageing-related processes, including antioxidant defence, anti-inflammatory signaling, preservation of mitochondrial function, activation of autophagy/mitophagy, and regulation of cell-cycle- and senescence-associated pathways. Representative upstream regulators and pathway nodes shown in the figure include SIRT1, AMPK, FOXO, Nrf2/ARE, SLC7A11/GPX4, NF-κB, NLRP3 inflammasome, mTORC1, PINK1/Parkin, ΔΨm, and so on; * indicates representative markers or context-dependent readouts rather than universally established direct mediators. Created with BioGDP.com [10]. The direction of the arrows indicates upregulation or downregulation).

Increasing evidence suggests that natural small molecules can delay ageing by maintaining cellular homeostasis. Their mechanisms not only involve the inhibition of oxidative stress but also encompass multiple oxidative stress-related synergistic antioxidant regulatory processes, such as the modulation of inflammatory responses, regulation of autophagy, and improvement of mitochondrial function (Figure 2).

It should be noted that the biological effects of medicinal plants may not arise entirely from a single natural monomer. In addition to organic bioactive constituents such as polyphenols, flavonoids, and alkaloids, trace elements contained in medicinal plants, including **zinc, copper, and selenium**, may also contribute in an auxiliary manner to the overall effects. These elements commonly serve as cofactors for various antioxidant and metabolic enzymes, and are involved in cellular metabolism, growth and development, immune responses, neural function, and reproductive processes, while also playing important roles in redox balance and the maintenance of physiological homeostasis [11]. These elements commonly serve as cofactors for various antioxidant and metabolic enzymes, and are involved in cellular metabolism, growth and development, immune responses, neural function, and reproductive processes, while also playing important roles in redox balance and the maintenance of physiological homeostasis [12]. Existing studies further suggest that some trace elements may be associated with UV damage responses, the regulation of signaling pathways such as MAPK and NF-κB [13], as well as collagen synthesis and the maintenance of fibroblast function [14]. Therefore, the overall anti-ageing effects of medicinal plants may not be attributable solely to a single natural monomer, and the trace elements they contain may also provide supportive contributions by helping maintain physiological homeostasis [15].

However, since the main focus of this review is to elucidate the molecular mechanisms of medicinal plant-derived natural monomers, trace elements are considered here only as auxiliary factors that may participate in the overall effects and are not discussed further in detail.

## 3. Anti-Ageing Mechanisms of Natural Small Molecules

### 3.1. Direct Antioxidant Effects

**Figure 3:**
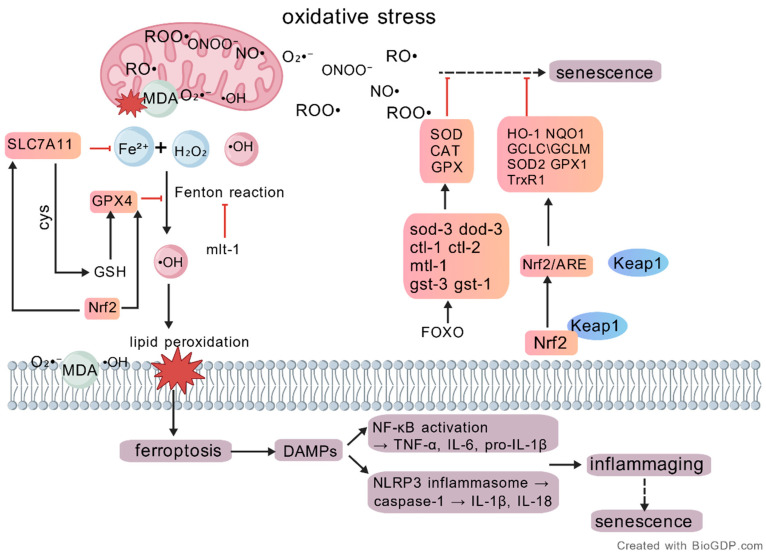
Focuses on oxidative stress-centred pathways linking ferroptosis-associated damage, inflammatory amplification, and senescence-related outcomes. (Oxidative stress may promote lipid peroxidation through iron-dependent radical generation and disruption of the SLC7A11–GSH–GPX4 antioxidant axis. Ferroptosis-associated cellular damage may lead to the release of damage-associated molecular patterns (DAMPs), which in turn may activate inflammatory pathways, including NF-κB-dependent transcription of TNF-α, IL-6, and pro-IL-1β, as well as NLRP3 inflammasome-mediated caspase-1 activation and maturation of IL-1β/IL-18, thereby contributing to inflammaging. Created with BioGDP.com [10]).

Oxidative stress represents a central driving force in the ageing process. Free radicals generated during oxidative stress directly accelerate ageing by inducing genetic mutations, protein denaturation, and lipid peroxidation through multiple mechanisms, including DNA damage, telomere shortening, mitochondrial dysfunction, and inflammatory responses. Under physiological conditions, cells depend on antioxidant systems (e.g., superoxide dismutase and catalase), which constitute a hierarchical scavenging network that eliminates free radicals and preserves cellular homeostasis (Figure 3). However, various physiological or pathological stimuli may disrupt the intracellular redox equilibrium, resulting in excessive free radical accumulation. This imbalance induces mitochondrial dysfunction, driving cells toward senescence or even apoptosis [16].

In response to oxidative stress, certain single-compound agents derived from natural herbal medicines may extend lifespan by enhancing the organism’s intrinsic resistance to oxidative stress [17]. However, most monomers derived from natural herbal medicines exert their effects primarily by restoring systemic redox balance, thereby mitigating oxidative damage and contributing to the attenuation of the ageing process. The specific mechanisms underlying these antioxidant effects are summarised as follows:

(1) Upregulating antioxidant enzymes

In the anti-ageing process, antioxidant enzymes (AOEs) play an important role in scavenging reactive oxygen species (ROS) and maintaining cellular redox homeostasis. Their expression is tightly regulated by several transcription factors, along with FOXO (DAF-16) and Nrf2, which are particularly important. DAF-16 is activated under conditions of diminished insulin signalling or caloric restriction. It regulates the transcription of genes such as sod-3 and ctl-1 to enhance the expression of downstream antioxidant enzymes, ctl-1 to promote downstream antioxidant enzyme expression [18]. Nrf2, conversely, binds to and activates the ARE (Antioxidant Response Element), driving the transcription of genes such as HO-1, NQO1, and GPx. This establishes a robust cellular antioxidant defence, effectively delaying the ageing process induced by oxidative stress [19].

Natural small molecules may attenuate ageing-related phenotypes and contribute to the management of age-related disorders by regulating antioxidant enzymes. For instance, naringin and hesperidin activate antioxidant enzyme systems, improving liver function in aged rats [20]. In recent years, an increasing number of natural small molecules have been reported to influence lifespan-related processes in preclinical models by regulating antioxidant enzymes via pathways such as the DAF-16 (FOXO) factor (Table 1 and Table 2). In mechanistic studies of natural small molecules delaying ageing, experimental evidence concerning the FOXO pathway has primarily focused on model organisms (e.g., Caenorhabditis elegans). Whilst some cellular and animal studies exist in mammalian systems, systematic research specifically addressing natural small molecule regulation of FOXO remains relatively limited. Therefore, although these findings are mechanistically informative, their translational relevance remains uncertain and further validation is required before clinical application can be considered.

Nrf2 is a transcription factor primarily regulating gene expression driven by the ARE. Under steady-state conditions, Nrf2 binds to the Keap1 protein and undergoes continuous degradation. Under stress conditions (such as ROS), Nrf2 dissociates, translocates into the nucleus, and initiates the transcription of antioxidant-related genes, activating the expression of multiple antioxidant enzymes and associated detoxification enzymes [40]. Galangin promotes the activation of downstream Nrf2 and HO-1 by activating upstream SIRT1 and PGC-1α, thereby counteracting ageing induced by oxidative damage [41]. Anthocyanins significantly extend Drosophila lifespan by downregulating Keap1 (an anti-longevity gene), activating Sirt6, and increasing Hif1 (a pro-longevity gene) expression, thereby enhancing antioxidant activity [42].

(2) Clearing MDA and Inhibiting Lipid Peroxidation

When oxidative stress becomes excessive, intracellular free radicals are not efficiently eliminated. Consequently, accumulated radicals attack essential biomolecules, including membrane lipids, proteins, and DNA, thereby impairing cellular integrity and accelerating the ageing process [16]. For example, free radicals peroxidise membrane lipids rich in polyunsaturated fatty acids, generating toxic aldehydes such as 4-hydroxy-2-nonenal and MDA—a process termed lipid peroxidation. These lipid aldehydes act as reactive signalling intermediates that alter protein conformation and function, thereby initiating a self-perpetuating lipid radical chain reaction leading to cellular degeneration. MDA, the terminal product of lipid peroxidation, is highly reactive and cross-links with DNA, proteins, and phospholipids to form cytotoxic adducts [43].

The removal of MDA does not occur via direct degradation but depends on endogenous antioxidant systems such as Nrf2, GSH, SOD, and GPx. For instance, ginsenoside Rg1 upregulates SOD and GSH-Px activities, reduces MDA accumulation, and thereby delays cellular ageing [44]. Other natural small molecules, including rutin [45] and paclitaxel [37], exert similar protective effects.

(3) Inhibition of Ferroptosis

Ferroptosis is a regulated form of cell death characterised by iron-dependent lipid peroxidation, with accumulation as its central mechanism [46]. During ferroptosis, damaged cells release damage-associated molecular patterns (DAMPs), which induce and amplify chronic inflammatory responses [47]. Meanwhile, chronic inflammation promotes ferroptosis by disrupting iron metabolism and increasing oxidative stress, thereby forming a vicious positive feedback loop between inflammation and ferroptosis [48].

Conventional antioxidants primarily target ROS to neutralise excess free radicals and prevent oxidative damage to cellular components. However, such agents often fail to prevent the activation of regulated cell death pathways. In contrast, ferroptosis inhibition aims to control the terminal phase of the lipid peroxidation cascade, specifically preventing cell death driven by iron accumulation and membrane lipid damage. Key molecular targets in ferroptosis include the glutathione peroxidase 4 (GPX4) enzyme, the SLC7A11 (xCT) cystine/glutamate transporter, and proteins involved in iron metabolism. These pathways collectively preserve lipid membrane integrity and prevent structural disruption caused by iron-dependent peroxidation reactions [49]. The FSP1–NADPH–CoQH_2_ axis serves as an independent secondary defence mechanism within the ferroptosis resistance network. By maintaining the antioxidant barrier of membrane lipids, this pathway prevents lipid peroxidation and provides an essential backup mechanism when GPX4 activity is compromised [50]. Ferritinophagy, a selective form of autophagy, is also recognised as a critical process in maintaining intracellular iron homeostasis [51].

Developing pharmacological inhibitors of ferroptosis to block lipid peroxidation and remove excess intracellular iron—thereby extending lifespan independently of other longevity pathways such as IGF-1—represents a promising future research direction [52]. Natural small molecules delay ageing and mitigate age-related diseases by modulating ferroptosis through multi-target and multi-level mechanisms. The principal mechanisms include: (1) activating the Nrf2/SLC7A11/GPX4 pathway to enhance antioxidant defence; (2) upregulating iron-storage proteins such as FTH1 and ferritin to limit Fe^2+^ participation in the Fenton reaction; and (3) improving energy metabolism and redox balance through the AMPK/Nrf2 or HDAC2/Nrf2/HO-1 signalling axes. Collectively, these actions preserve iron homeostasis and mitochondrial integrity, thereby mitigating lipid peroxidation-induced damage and contributing to anti-ageing effects. The specific mechanisms are outlined below (Table 3).

(4) Regulation of Upstream Antioxidant Factors

The sirtuin (SIRT) protein family represents a well-established group of longevity-associated genes. As a family of NAD^+^-dependent deacetylases, it consists of seven members (SIRT1–SIRT7) that are distributed across various cellular compartments—including the nucleus, cytoplasm, and mitochondria—where they play essential roles in regulating ageing, metabolism, antioxidant defence, and DNA repair [60]. Among them, SIRT1 is the most extensively studied and participates in multiple biological processes associated with ageing, including the regulation of chronic inflammation, maintenance of mitochondrial function, activation of autophagy, and modulation of apoptosis, underscoring its pivotal role in the anti-ageing network. The multifaceted mechanisms through which SIRT1 regulates ageing, as well as recent research progress, are summarised below. First, the antioxidant effects of key natural small molecules acting through SIRT1 are discussed. Natural small molecules exert antioxidant effects primarily through SIRT1-mediated deacetylation mechanisms. On one hand, they enhance Nrf2 activity via deacetylation, which strengthens its transcriptional regulation of antioxidant target genes such as HO-1, NQO1, and SOD, thereby improving cellular antioxidant defences [61]. On the other hand, SIRT1 deacetylates FOXO transcription factors, leading to the upregulation of antioxidant enzymes and enhanced cellular protection [62]. Additionally, natural small molecules enhance metabolic activity by regulating PGC-1α- and HIF-1α-related genes, promoting mitochondrial biogenesis and energy metabolism, thereby increasing stress resistance and extending lifespan [63] (Table 4).

### 3.2. Indirect Antioxidant Effects

During the progression of ageing, oxidative stress is not merely a single damaging event, but rather a systemic “amplifier” that spans multiple biological modules: the continuous accumulation of ROS/RNS triggers and reshapes key biological processes, forming self-reinforcing feedback loops with these processes, thereby accelerating functional decline and multi-organ ageing. The core concept is that oxidative stress acts as an upstream trigger for molecular damage and signaling abnormalities, and, through its effects on energy metabolism, protein homeostasis, inflammatory networks, and gene expression programs, further elevates the oxidative load in cells and tissues, ultimately leading to an irreversibly altered ageing state.

Firstly, at the autophagy/mitophagy level, ROS can serve as an acute stress signal to induce autophagy initiation, which is a protective adaptive response to clear oxidative damage proteins and dysfunctional organelles [67]. However, during ageing, the autophagic flux and lysosomal function often decline, leading to insufficient clearance of damaged mitochondria and protein aggregates, which in turn causes ROS levels to persistently rise and further inhibit autophagic efficiency, creating a positive feedback loop of “oxidative stress–autophagic decline–damage accumulation.” Particularly, insufficient mitophagy results in the accumulation of low-quality mitochondria, shifting oxidative stress from a “reversible response” to a chronic state of “increased set point” [68].

Secondly, at the mitochondrial function level, mitochondria are both a major source of endogenous ROS and a primary target of oxidative damage. Proteins in the electron transport chain, mitochondrial membrane lipids (e.g., cardiolipin), and mtDNA are highly sensitive to oxidative stress. Once damaged, electron transfer efficiency declines, electron leakage increases, leading to further ROS accumulation and the formation of a classic “mitochondrial dysfunction–ROS elevation” vicious cycle [69]. This loop not only accelerates cellular energy crises but also promotes the release of mitochondrial damage-associated molecular patterns (DAMPs), triggering subsequent inflammatory amplification.

Thirdly, there is a close coupling between oxidative stress and chronic inflammation [70]. Oxidative products, mtDNA leakage, and mitochondrial damage-related signals can activate inflammasomes (e.g., NLRP3) and NF-κB pathways, promoting the release of inflammatory mediators like IL-1β, IL-6, and TNF. In turn, inflammatory cells and enzymes (e.g., NOX and iNOS) continue to generate ROS/RNS, exacerbating the oxidative load, thus forming the classic “inflammation–oxidation” bidirectional amplification loop. This loop results in a microenvironment that remains under chronic low-grade inflammation and oxidative stress at the tissue level, providing a common ground for the degeneration of multiple systems related to ageing.

Fourth, oxidative stress induces DNA damage, triggering the DNA damage response (DDR) and influencing cell cycle fate [67]. ROS can induce nuclear DNA and mtDNA damage, replication stress, and telomere dysfunction (such as accelerated telomere shortening), activating the p53/p21 and p16/Rb axes, leading to cell cycle arrest and cellular senescence.

When the damage becomes more severe or repair fails, it progresses to apoptosis mediated by mitochondria. It is noteworthy that senescent cells often accompany persistent oxidative stress and mitochondrial dysfunction, and through SASP secretion, further drive local inflammation and bypass ageing, expanding oxidative stress from an intracellular event to a tissue-level “propagating” ageing mechanism.

Finally, in epigenetic regulation, oxidative stress not only causes DNA base oxidation and chromatin structural disruption but also affects methylation/demethylation processes and histone modification enzyme activities, driving gene expression drift and increasing “epigenetic noise.” Meanwhile, the epigenetic state, in turn, regulates the expression thresholds of antioxidant defense, mitochondrial homeostasis, and autophagy-related genes (e.g., Nrf2 pathway and its downstream antioxidant enzymes), thus resetting the “baseline” of redox homeostasis on a longer timescale [71]. This creates a slowly changing feedback between oxidative stress and epigenetics: short-term stress can be buffered, but long-term accumulation will solidify into stable ageing transcription programs.

**Figure 4:**
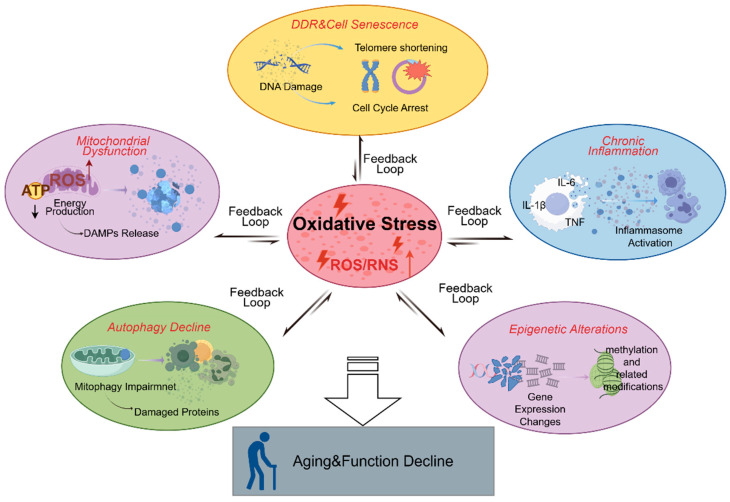
Oxidative stress as a central hub connecting multiple ageing-related processes. (Oxidative stress induced by the excessive accumulation of reactive oxygen species/reactive nitrogen species (ROS/RNS) interacts with multiple ageing-related processes, including mitochondrial dysfunction, the DNA damage response (DDR) and cellular senescence, chronic inflammation, epigenetic alterations, and impaired autophagy. These processes may form mutually amplifying feedback loops, ultimately leading to ageing and functional decline.)

In summary, oxidative stress, autophagy/mitophagy, mitochondrial function, chronic inflammation, DDR-cell fate, and epigenetic reprogramming together form a nested feedback network (Figure 4). This suggests that anti-ageing interventions should not be limited to “directly scavenging free radicals.” Instead, strategies could involve enhancing mitochondrial quality control, increasing autophagic flux, suppressing inflammatory cascades, and remodeling redox-related transcriptional programs, thereby indirectly reducing oxidative load and interrupting positive feedback loops, which in turn may systemically delay ageing and age-related diseases. With its multi-target regulatory advantages, natural small molecules not only possess direct antioxidant effects but also can alleviate other ageing-related pathological changes induced by oxidative stress, thereby further delaying the ageing process.

### 3.3. Activation of Autophagy and Mitochondrial Protection

**Figure 5:**
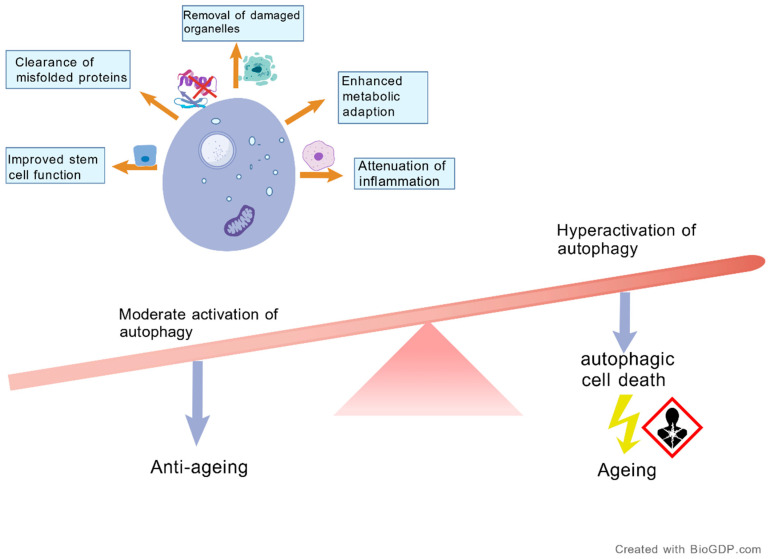
The dual role of autophagy in ageing: moderate activation is protective, whereas excessive activation may be detrimental. (Intensity-dependent effects of autophagy in ageing. Moderate activation of autophagy exerts anti-ageing effects by promoting the clearance of misfolded proteins, removing damaged organelles, improving stem cell function, enhancing metabolic adaptability, and alleviating inflammation. In contrast, excessive or persistently high-level activation of autophagy may induce autophagic cell death and aggravate ageing-related damage. Created with BioGDP.com [10]).

Autophagy preserves cellular homeostasis by degrading damaged organelles and macromolecules in lysosomes, thereby reducing oxidative stress, removing inflammatory mediators, and regulating innate immunity to delay ageing and maintain cellular and tissue integrity [72]. In elderly individuals, impaired autophagy disrupts homeostatic balance, leading to chronic inflammation and accelerated ageing [73]. Under certain stress conditions, excessive activation of autophagy can induce cellular senescence and exert detrimental effects. Therefore, moderate inhibition of autophagy may help delay the ageing process [74] (Figure 5).

**Figure 6:**
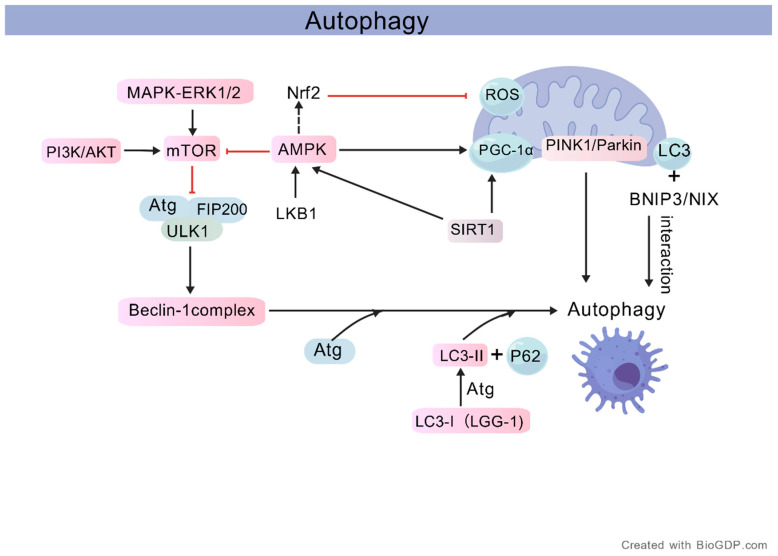
Major signaling pathways involved in the regulation of autophagy and mitophagy in the anti-ageing context. (Representative signaling pathways related to autophagy and mitophagy. The PI3K/AKT and MAPK–ERK1/2 pathways converge on mTOR, which negatively regulates the autophagy initiation machinery, including Atg proteins, FIP200, ULK1, and the Beclin-1 complex. AMPK can be activated by the upstream kinase LKB1, thereby promoting autophagy, and is also linked to Nrf2 and PGC-1α. The major mitophagy-related pathways include PINK1/Parkin and BNIP3/NIX signalling, both of which are closely associated with mitochondrial quality control. LC3, LC3-II, p62, and Atg proteins represent key components involved in autophagosome formation and degradation. In the figure, LGG-1 denotes the Caenorhabditis elegans homolog of LC3. Created with BioGDP.com [10]).

Mitophagy, a form of selective autophagy, recognises and removes damaged mitochondria to maintain mitochondrial quality control and intracellular homeostasis (Figure 6). Reduced mitophagic activity is closely associated with the onset and progression of numerous age-related diseases [75].

Natural small molecules can activate autophagy to delay ageing (Table 5). More specifically, the anti-ageing effects of autophagy and mitophagy are not merely reflected by enhanced degradation, but rather depend on a series of well-defined processes involving signal regulation, gene-expression reprogramming, and organelle quality control. Under conditions of nutrient sufficiency or active pro-growth signaling, PI3K/AKT and MAPK–ERK1/2 activate mTOR, which in turn suppresses the ULK1/FIP200 autophagy initiation complex, thereby limiting Beclin-1 complex-mediated phagophore nucleation and subsequent ATG-dependent autophagosome formation. In contrast, under energy stress conditions, LKB1 activates AMPK, while SIRT1 can also enhance metabolic adaptation and mitochondrial quality control by promoting AMPK and PGC-1α activity. On the one hand, AMPK counteracts mTOR-mediated inhibition of autophagy; on the other hand, it promotes the recognition and clearance of damaged mitochondria through PGC-1α and the PINK1/Parkin pathway. Meanwhile, BNIP3/NIX, as receptors located on the outer mitochondrial membrane, can interact with LC3 and further mediate selective mitophagy. During the execution phase of autophagy, ATG family proteins promote the lipidation-dependent conversion of LC3-I to LC3-II, while LC3-II, together with p62/SQSTM1, participates in the recruitment of damaged cargo and autophagosome maturation. Through these processes, cells can effectively eliminate damaged mitochondria and abnormal protein aggregates generated under excessive ROS production, thereby alleviating oxidative stress and chronic inflammation, limiting mtDNA oxidative damage and its leakage into the cytoplasm, and indirectly reducing the burden of nuclear DNA damage and persistent activation of the DNA damage response. Thus, autophagy/mitophagy not only maintains metabolic and redox homeostasis, but also promotes the preservation of both nuclear and mitochondrial genome stability by limiting persistent upstream stimulation of senescence effector pathways such as p53–p21 and p16. Accordingly, the anti-ageing effects of natural monomeric compounds are reflected not only in the maintenance of metabolic and redox homeostasis, but also in the coordinated preservation of nuclear and mitochondrial genomic stability.

### 3.4. Maintaining Mitochondrial Function

Excessive accumulation of reactive oxygen species (ROS) can induce mitochondrial damage and accelerate mitochondrial senescence, while simultaneously causing oxidative damage to DNA, proteins, and lipids. This oxidative stress activates key senescence-associated signalling pathways, including p53, p21, and p16, while triggering chronic low-grade inflammation that promotes the onset and progression of programmed cellular senescence [82]. At the same time, impaired mitochondrial energy metabolism compromises the function of high-energy-demand tissues such as the heart, brain, and skeletal muscle, and weakens metabolic and signalling regulation in immune cells. These dysfunctions ultimately accelerate organ ageing and contribute to the progression of age-related diseases [83]. In addition to reducing mitochondrial oxidative stress, specific natural small molecules can modulate mitochondrial dysfunction and delay ageing driven by mitochondrial senescence through several mechanisms (Table 6).

(1) Maintaining Mitochondrial Oxidative Phosphorylation (OXPHOS)

OXPHOS is essential for sustaining cellular energy homeostasis. Defects in OXPHOS lead to overproduction of ROS, resulting in DNA damage and epigenetic alterations, and may indirectly accelerate telomere shortening, thereby promoting ageing-related phenotypes [92]. In multiple longevity models—such as daf-2 mutants, dietary restriction, cold exposure, or partial inhibition of the electron transport chain—mitochondrial dynamics are remodelled, often characterised by enhanced fusion that correlates with extended lifespan [93]. Preserving OXPHOS integrity helps maintain mitochondrial membrane potential and ATP production, thereby supporting normal mitochondrial dynamics [94].

(2) Stabilising Mitochondrial Membrane Potential

The stability of the mitochondrial membrane potential (ΔΨm) does not depend solely on electron transport chain activity, but is jointly maintained by mitochondrial energy metabolism, ionic homeostasis, redox balance, quality control, and NAD+-dependent regulation. First, electron transport chain complexes I, III, and IV establish ΔΨm by generating a proton gradient across the inner mitochondrial membrane [95]. In addition, disturbances in Ca^2+^ homeostasis, adenine nucleotide transport, and mitochondrial membrane permeability—particularly the abnormal opening of the mitochondrial permeability transition pore (mPTP)—can lead to ΔΨm collapse, ATP depletion, and aberrant mtDNA leakage. At the level of gene expression, antioxidant transcriptional programmes mediated by NRF2 and FOXO3 can upregulate genes such as HMOX1, NQO1, GCLC, GCLM, SOD2, GPX1, and TXNRD1, thereby limiting ROS-mediated membrane lipid peroxidation and mtDNA damage [96]. In addition, PINK1/Parkin-dependent and BNIP3/NIX-dependent mitophagy selectively remove damaged mitochondria with reduced membrane potential and excessive ROS, thereby preventing the accumulation of dysfunctional mitochondria [97]. Moreover, a decline in NAD+ levels weakens SIRT1 activity and reduces the ability of PGC-1α to support mitochondrial biogenesis and metabolic reprogramming, which is unfavorable for maintaining tricarboxylic acid cycle flux, oxidative phosphorylation, and redox homeostasis [85].

Together, these mechanisms maintain ΔΨm stability, sustain cellular energy supply and viability, and are closely linked to the progression of ageing [98].

(3) Delaying Immunosenescence

With advancing age, T cells commonly exhibit mitochondrial dysfunction, abnormal membrane potential, and reduced metabolic adaptability, which in turn impair their stem-like characteristics, proliferative potential, and effector functions. At the same time, the age-related decline in NAD levels is also considered an important factor contributing to mitochondrial dysfunction in aged T cells [99]. Therefore, immunosenescence is not only reflected by elevated inflammatory status, but is also accompanied by disrupted energy metabolism in immune cells and a reduced capacity to maintain intracellular homeostasis. Further studies have shown that T-cell functional decline is often accompanied by reduced levels of autophagy. When autophagy is insufficient, CD8 T cells fail to remove damaged mitochondria in a timely manner and may abnormally inherit aged mitochondria during cell division, thereby impairing memory differentiation potential and disrupting metabolic programming [100]. Thymic involution and the persistent decline in naïve T cells are important structural features of immunosenescence; age-related immune remodeling may lead to functional skewing of memory T cells and further affect B-cell responses and vaccine efficacy.

Current evidence suggests that restoring NAD homeostasis and enhancing autophagy/mitophagy may help improve mitochondrial quality control, metabolic flexibility, and memory differentiation capacity in aged T cells, thereby delaying the progression of immunosenescence to some extent [101]. Urolithin A, for example, has been reported to increase naïve CD8^+^ T cells and remodel immunometabolism by promoting mitophagy, thereby counteracting age-related immune decline [102]. Certain polyphenols may also ameliorate immunosenescence-related phenotypes to some extent; for instance, EGCG has been reported to alleviate murine immunosenescence and inflamm-aging [103], while curcumin can reduce the proportion of PD-1^+^ cytotoxic T cells in aged mice and improve hematopoietic stem cell function [104].

### 3.5. Effects on Alleviating Chronic Inflammation

**Figure 7:**
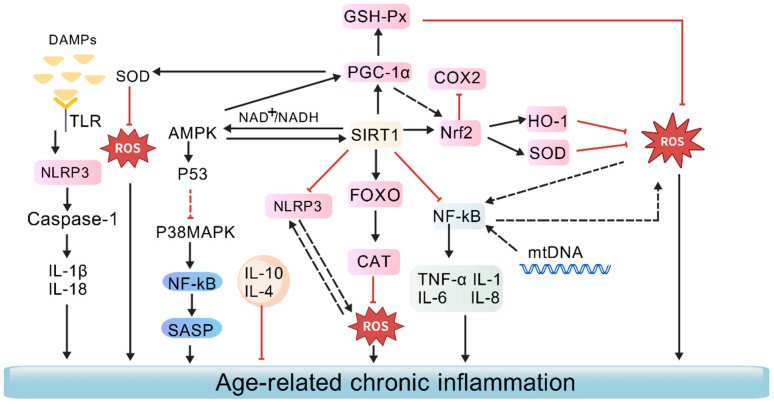
Interaction between oxidative stress and age-related chronic inflammation. (Bidirectional interplay between oxidative stress and chronic inflammation during ageing. Damage-associated molecular patterns (DAMPs) can activate Toll-like receptors (TLRs), thereby promoting NLRP3 inflammasome activation, caspase-1 cleavage, and the release of pro-inflammatory cytokines such as IL-1β and IL-18. ROS can also promote the activation of p38MAPK, NF-κB, and the senescence-associated secretory phenotype (SASP). Meanwhile, pathways or molecules such as AMPK, SIRT1, PGC-1α, Nrf2, FOXO, SOD, CAT, GSH-Px (GPx), and HO-1 represent antioxidant and anti-inflammatory regulatory mechanisms. In addition, mitochondrial DNA (mtDNA) release and inflammatory mediators such as TNF-α, IL-1, IL-6, and IL-8 further amplify age-related chronic inflammation. Created with BioGDP.com [10]).

With advancing age, the body exhibits a persistent, low-grade systemic inflammatory state. Even without evident infection, persistent elevation of inflammatory cytokines such as IL-6, TNF-α, and CRP is commonly observed. This chronic inflammatory state damages cells and tissues, promotes DNA damage, protein misfolding, and mitochondrial dysfunction, and thereby accelerates the ageing process [6] (Figure 7). In addition, during ageing, there is a bidirectional reinforcing relationship between infection, chronic inflammatory diseases, and immunosenescence: immunosenescence weakens pathogen clearance and immune surveillance, thereby increasing susceptibility to infections and chronic inflammatory diseases; conversely, persistent infection and inflammation can further accelerate the progression of immunosenescence by promoting immune-cell exhaustion, mitochondrial dysfunction, and amplification of inflammatory signaling.

Meanwhile, senescent cells release the senescence-associated secretory phenotype (SASP), a cascade composed of pro-inflammatory cytokines, chemokines, proteases, and some growth factors. Common components include IL-6, IL-1β, IL-8, TNF-α, MCP-1/CCL2, and MMPs, which can drive the onset and persistence of chronic low-grade inflammation (inflammaging). Senescent cells release the senescence-associated secretory phenotype (SASP), a cascade composed of pro-inflammatory cytokines, chemokines, proteases, and some growth factors. Common components include IL-6, IL-1β, IL-8, TNF-α, MCP-1/CCL2, and MMPs, which drives the initiation and persistence of chronic low-grade inflammation (inflammaging). Among these, matrix metalloproteinases (MMPs), as important proteolytic components, promote the degradation of collagen and other extracellular matrix constituents, leading to tissue structural disruption and microenvironmental homeostatic imbalance [105]. In addition, inflammation-related effector enzymes such as COX-2 and iNOS, although not cytokines or proteases themselves, can amplify inflammatory responses and aggravate oxidative/nitrosative damage by promoting prostaglandin production and high-output nitric oxide release, respectively, thereby jointly contributing to ageing-related degenerative tissue changes [106].

During this process, reactive oxygen species (ROS) enhance NF-κB-dependent protein ubiquitination and proteasomal degradation, which in turn stimulate pro-inflammatory cytokine production and catabolic activity. In addition, ROS can further promote extracellular matrix degradation, inflammatory amplification, and tissue injury by activating stress- and inflammation-related pathways such as MAPK, NF-κB, and AP-1, thereby upregulating the expression of MMPs, COX-2, and NOS [107]. Conversely, promoting eNOS expression or phosphorylation, reducing ROS-mediated scavenging of NO, and downregulating vasoconstrictive factors such as endothelin-1 (ET-1) may help improve endothelial ageing.

Elevated pro-inflammatory mediators, including IL-1β, IL-6, CRP, TNF-α, and IFN-γ, exacerbate tissue damage and amplify paracrine inflammatory signalling, thereby accelerating ageing [70]. The NLRP3 inflammasome serves as a central regulator of chronic inflammation; its activation leads to the cleavage and activation of Caspase-1. Activated Caspase-1 then cleaves pro-forms of inflammatory cytokines, facilitating their release and amplifying inflammatory responses [108]. Reducing mitochondrial membrane permeability or removing cytosolic mtDNA (mitochondrial DNA) markedly decreases SASP levels, indicating that mtDNA leakage acts as a major driver of SASP formation [109]. Oxidised mitochondrial DNA fragments released through the mPTP (mitochondrial permeability transition pore)/VDAC (voltage dependent anion channel) pore can activate NLRP3 inflammasomes, a process recognised as integral to the ageing phenotype [110]. In addition, activation of the TLR4/NF-κB pathway and the NLRP3 inflammasome may serve as important mechanistic bases through which gut microbiota dysbiosis promotes ageing. Extensive studies demonstrate that specific natural small molecules attenuate chronic inflammation through multiple molecular pathways, thereby contributing to delayed ageing (Table 7) (Figure 7). SIRT1 further regulates inflammation by modulating the acetylation of NF-κB p65 [111] and controlling the transcription of genes encoding inflammatory mediators, including IL-1, TNF-α, IL-8, and IL-6 [112], thereby exerting potent anti-ageing effects (Table 8).

### 3.6. Increasing Heat Stress Resistance and Maintaining Protein Homeostasis

Imbalance in protein homeostasis is recognized as one of the twelve classical hallmarks of ageing. Heat stress disrupts intracellular proteostasis and promotes the generation of reactive oxygen species (ROS) by inducing mitochondrial dysfunction and protein misfolding. Excessive ROS accumulation further aggravates cellular damage and reduces cellular tolerance to heat stress, thereby forming a positive feedback loop that amplifies stress responses and accelerates cellular ageing. In contrast, activation of heat shock proteins (HSPs) restores proteostasis by facilitating the refolding or degradation of misfolded proteins, thereby alleviating ageing-related cellular damage. Natural small molecules enhance cellular heat stress resistance primarily by activating the HSF1 (heat shock transcription factor 1) signalling pathway, which in turn induces the expression of HSPs. For instance, nobiletin [24], Hyperoside [130], and paeonol [23] have been shown to activate HSF1 and upregulate HSP expression. These effects are frequently accompanied by the intrinsic antioxidant activities of natural small molecules. Together, these mechanisms constitute an essential component of the cellular defence system against ageing, preserving cellular function and delaying the progression of the ageing process.

### 3.7. Regulation of the Cell Cycle

Oxidative stress induces DNA damage and activates the p53/p21 and p16/Rb signaling pathways, which inhibit cyclin-dependent kinase (CDK) activity and consequently lead to cell cycle arrest. In the short term, this process helps maintain genomic stability, and may be associated with acute senescence, a protective cellular response; in contrast, persistent oxidative stress may drive it toward irreversible arrest and further promote its progression into pathological chronic senescence. Therefore, the anti-ageing effects of natural small molecules should be understood primarily as the regulation of pathological chronic senescence.

(1) Regulating Apoptosis

Oxidative stress is closely associated with apoptosis. Excessive reactive species (ROS/RNS) can trigger mitochondrial-dependent apoptosis by inducing mitochondrial membrane damage, activating Bax/BAK, promoting cytochrome c release, and initiating the caspase cascade [131]. Maintaining appropriate apoptotic clearance also helps limit the persistence of chronic inflammation. When aged or damaged cells fail to undergo timely apoptotic clearance, they persist and secrete SASP factors, thereby perpetuating chronic inflammation. Beyond this, the ultimate role of apoptosis regulation is to preserve tissue homeostasis and functional integrity. Chronic mitochondrial apoptotic stress may not immediately induce cell death; rather, it can trigger sublethal signaling events that lead to cell cycle arrest and cellular senescence—one of the critical upstream events in ageing [109]. Furthermore, in senescent cells exposed to apoptotic stress, cytoplasmic release of mtDNA activates the cGAS–STING pathway. This activation induces the transcription of inflammation-associated genes and promotes the secretion of SASP components such as IL-6 and IL-8, thereby exacerbating cellular senescence. In other words, moderate suppression of pro-apoptotic proteins (e.g., Bax, Caspase-3) or upregulation of anti-apoptotic proteins (e.g., Bcl-2, Bcl-xL, Mcl-1) may represent effective strategies for delaying tissue ageing [132].

Notably, apoptosis-related factors exhibit bidirectional regulatory effects on cellular senescence. For example, Bcl-2 supports the survival of senescent cells by suppressing apoptosis but, under certain conditions, can paradoxically promote senescence through upregulation of CDK inhibitors (p27, Rb family member p130) and downregulation of E2F target genes [132]. This suggests that, in the regulation of ageing, the key issue is not simply whether apoptosis occurs, but whether the apoptosis–survival balance is disrupted (Table 9). Therefore, a more rational intervention strategy is not to indiscriminately suppress or enhance apoptosis in a unidirectional manner, but rather to reduce pathological excessive apoptosis caused by persistent stress while preserving the appropriate clearance of abnormal cells, thereby maintaining tissue homeostasis. This should be distinguished from the situation in tumor cells, where ROS imbalance and apoptotic escape often act together to promote tumor progression [133]. This also implies that apoptosis should not be suppressed indiscriminately, because the timely elimination of abnormal cells or potentially malignant transformed cells is equally important.

However, existing studies on the anti-ageing effects of specific natural small molecules indicate that apoptosis modulation often occurs concurrently with other processes, such as suppression of chronic inflammation or regulation of the cell cycle. Direct evidence demonstrating that these compounds delay ageing primarily through apoptosis regulation remains limited. Therefore, this review does not further elaborate on this mechanism.

(2) Modulating Pathological Cell Cycle Arrest

**Figure 8:**
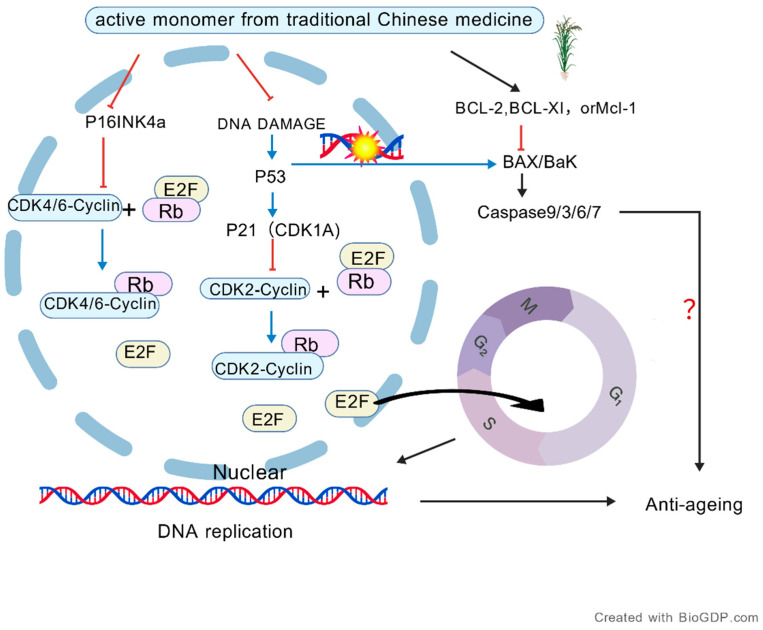
Representative pathways through which bioactive monomers from traditional Chinese medicine regulate cell-cycle arrest and apoptosis in the anti-ageing context. (This figure summarizes the representative relationships among cell-cycle regulation, DNA damage signaling, and apoptosis-related pathways. DNA damage can activate p53, which in turn induces p21 (CDKN1A) expression and inhibits the activity of CDK–cyclin complexes. Meanwhile, p16^INK4a suppresses CDK4/6–cyclin activity. Together, these changes regulate the phosphorylation status of Rb and the release or inhibition of the E2F transcription factor, thereby affecting DNA replication and cell-cycle progression through the G1/S/G2/M phases.In the apoptosis module, anti-apoptotic proteins such as BCL-2, BCL-XL, and Mcl-1 counteract pro-apoptotic proteins BAX/BAK and the downstream activation cascade of caspase-9/3/6/7. This figure is intended to provide a representative mechanistic framework through which bioactive monomers from traditional Chinese medicine may regulate ageing-related cell-cycle arrest and excessive apoptosis. Created with BioGDP.com [10]).

Mitochondrial apoptosis-induced cellular senescence represents a stress response characterised by irreversible cell cycle arrest, and this process is considered to be closely associated with one of the major hallmarks of ageing [140]. This process is frequently driven by the combined effects of telomere attrition, oxidative stress, DNA damage, and chromatin remodelling. Within this context, cell cycle signalling plays a pivotal regulatory role. Targeted modulation of the p16/p21/p53 axis and activation of CDK–Cyclin complexes enable precise intervention in cell cycle progression, thereby delaying the onset of cellular senescence. This strategy holds substantial potential for both basic and translational ageing research (Figure 8).

Existing studies indicate that medicinal plant-derived natural small molecules can influence the ageing process by regulating relevant cell-cycle factors. Their effects are mainly manifested in alleviating persistent pathological cell-cycle arrest, reducing the accumulation of senescent cells, and maintaining tissue homeostasis. The related mechanisms are summarized in Table 9.

Although current research on individual natural small molecules in this field remains limited, future advancements—including deeper mechanistic investigations, systematic compound screening, and the development of multi-target therapeutic strategies—may offer novel avenues for treating age-related diseases. Such approaches could ultimately improve clinical outcomes and enhance quality of life in ageing populations.

### 3.8. Reversing or Delaying Telomere Attrition

Telomeres gradually shorten during successive cell divisions, and once they reach a critical length, cellular senescence is triggered, leading to a decline in cellular function. Oxidative stress accelerates telomere shortening and suppresses telomerase activity. As telomeres progressively shorten, the proliferative capacity of stem cells decreases, tissue repair becomes impaired, and these changes are closely associated with various age-related degenerative diseases [141]. Telomerase counteracts telomere loss during DNA replication by maintaining telomere length, thus preserving genomic stability and mitigating age-related physiological deterioration.

Although telomere shortening may, to some extent, help restrict the proliferation of abnormal cells, persistent telomere attrition and the resulting genomic instability may also increase the risk of tumorigenesis. Therefore, from an anti-ageing perspective, a more important goal is to maintain telomerase activity within a safe range so as to stabilize telomere length, preserve stem cell function, and delay the ageing process.

Cycloastragenol activates telomerase, delays telomere shortening, and alleviates ageing-related impairment of follicular development [142]. Astragaloside IV similarly exerts anti-ageing effects by activating telomerase, thereby significantly reducing the proportion of senescent cells [143]. Furthermore, curcumin, quercetin, and resveratrol exhibit anti-ageing properties by activating telomerase and modulating additional longevity-related pathways [144]. Similarly, oleanolic acid and kaempferol demonstrate significant anti-ageing potential through telomerase-mediated pathways [145].

### 3.9. Regulation of Epigenetic Modifications

Epigenetic modifications play a fundamental role in the ageing process and may represent a pivotal breakthrough in anti-ageing research [71]. In ovarian ageing cell models, resveratrol significantly ameliorates transcriptional alterations associated with ageing. This effect primarily depends on Tet2-mediated DNA demethylation, which reprograms the methylome and restores normal gene expression to maintain a youthful transcriptional state in oocytes. This effect is particularly evident in middle-aged oocytes, thereby contributing to the delay of ovarian ageing [146]. It should be noted that ageing-related epigenetic alterations extend beyond DNA methylation to include histone modifications, chromatin remodelling, non-coding RNAs (ncRNAs), and RNA modifications. Meanwhile, microRNAs (miRNAs), as important post-transcriptional regulatory elements, are involved not only in the regulation of cellular senescence, SASP, and metabolism/longevity-related pathways, but may also serve as potential targets and tools in anti-ageing interventions [147].

Current evidence suggests that some natural monomeric compounds may participate in ageing regulation through miRNA-related signaling axes; however, studies in this direction remain relatively limited and are mainly concentrated in preclinical models. Representative examples include resveratrol, which alleviates airway epithelial cell senescence through the miR-34a/SIRT1/NF-κB axis [148], and EGCG, which may also improve senescence-like cellular phenotypes by modulating miRNA expression profiles [11].

Although current research on individual Chinese herbal monomers remains limited, their potential roles warrant further investigation and may offer promising directions for future anti-ageing strategies.

### 3.10. Calorie Restriction Mimicry

Calorie restriction (CR) represents one of the most extensively validated strategies for delaying ageing and extending lifespan. It involves a reduction in total energy intake by approximately 20–40% without inducing malnutrition and has demonstrated broad anti-ageing benefits across diverse organisms, including yeast, nematodes, fruit flies, rodents, and primates. Early calorie restriction mimetics (CRMs) were identified as compounds capable of promoting protein deacetylation and stimulating autophagy, thereby reproducing the biochemical and functional outcomes of CR [149]. As early as 2014, several natural autophagy activators were identified, including epigallocatechin gallate (EGCG) and curcumin [150]. Subsequent studies have shown that the anti-ageing effects of CRMs are mediated through multiple synergistic pathways, including energy sensing, antioxidant defence, mitochondrial regulation, inflammation suppression, and DNA repair. Autophagy constitutes an essential component rather than the sole mechanism underlying these effects.

In addition to activating autophagy to delay ageing, natural small molecules can mimic CR to exert anti-ageing effects and alleviate age-related degenerative changes through several mechanisms (Table 10).

Among the various anti-ageing interventions, CR has consistently been demonstrated to be among the most effective and reliable approaches. However, long-term and strict CR poses significant challenges in clinical practice, including poor compliance and potential nutritional deficiencies. Therefore, researchers have explored combinatorial strategies integrating natural small molecules with CR. Experimental evidence indicates that natural small molecules possessing antioxidant, anti-inflammatory, and epigenetic regulatory activities can markedly potentiate the effects of CR on metabolic homeostasis, mitochondrial autophagy, and inflammation control, thereby producing more pronounced anti-ageing outcomes in animal models [157]. This strategy offers novel insights into the mechanisms of natural small molecules and paves the way for developing feasible and effective anti-ageing interventions. Future clinical investigations are needed to systematically validate combined interventions involving various natural small molecules and CR, and to further evaluate their safety and translational potential.

### 3.11. Endocrine Ageing

The endocrine ageing theory proposes that, with advancing age, the regulatory function of the hypothalamic–pituitary–peripheral target gland axis gradually declines, leading to changes in the secretion levels, rhythms, and target tissue responsiveness of multiple hormones. These alterations subsequently affect energy metabolism, immune homeostasis, redox balance, tissue repair, and cell fate regulation, thereby promoting organismal ageing. Age-related changes in insulin/IGF-1, growth hormone, sex hormones, melatonin, and glucocorticoids are all considered to be closely associated with ageing phenotypes and age-related diseases [158]. Compared with oxidative stress- or inflammation-related regulation, medicinal plant-derived natural monomers whose anti-ageing effects are mainly mediated through “endocrine ageing/hormonal regulation” appear to be relatively limited, with representative compounds being concentrated mainly among phytoestrogens. Genistein is one of the more representative isoflavones in this category. It can influence skin homeostasis and other ageing-related phenotypes through estrogen receptor-related pathways [159], and serves as a typical example of how natural small molecules may participate in anti-ageing effects through hormone-like regulation.

## 4. Application of Natural Small Molecules in Anti-Ageing Clinical Research

At present, the clinical application of medicinal plant-derived natural small molecules in anti-ageing remains limited. Although clinical research in this field is gradually increasing, most available evidence is still preclinical, while human studies remain relatively scarce and are generally limited to early-phase or exploratory investigations.

Although clinical research on natural small molecules in the field of anti-ageing is gradually increasing, such as the testing of dasatinib and quercetin in early-phase studies, their actual clinical translation remains insufficient. Therefore, the clinical use of natural small molecules represents a major gap in current research, highlighting the need for high-quality clinical trials to confirm their efficacy and safety in anti-ageing therapy.

The regulatory effects of natural monomeric compounds on ageing-related mechanisms may, to some extent, be collectively reflected in improvements in stem cell homeostasis and the stem-cell microenvironment, thereby helping to preserve tissue regenerative capacity and attenuate ageing-related functional decline. Stem cells play a central role in tissue regeneration by replenishing damaged cells through self-renewal and directed differentiation, while also participating in tissue repair and reconstruction through regulation of the local microenvironment, inflammatory responses, and angiogenesis. Therefore, stem cell homeostasis and the condition of the stem-cell niche are major determinants of regenerative capacity. The regulatory effects of natural monomeric compounds on ageing-related mechanisms—including oxidative stress, inflammation, autophagy, mitochondrial homeostasis, and epigenetic regulation—may ultimately converge, at least in part, on improving stem cell function and the stem-cell microenvironment, thereby preserving tissue regenerative capacity and slowing ageing-related functional decline.

## 5. Comparison of Medicinal Plants-Derived Bioactive Compounds with Modern Pharmaceuticals

Natural small molecules and modern pharmaceuticals differ substantially in their chemical composition, mechanisms of action, clinical use, and safety profiles, each offering distinct advantages and limitations. Natural small molecules are typically derived from natural plant, animal, or mineral sources, characterised by complex compositions and relatively mild effects, often exerting multi-target regulatory actions. By contrast, modern drugs are mostly synthetic or semi-synthetic, with clearly defined molecular structures, strong target specificity, and rapid pharmacological action, though they often produce more significant side effects.

Mechanistically, modern pharmaceuticals generally act on single molecular targets. For example, metformin regulates glucose metabolism primarily through AMPK activation, whereas rapamycin modulates ageing by inhibiting mTOR. In contrast, natural small molecules generally exhibit multi-target regulatory activity. For instance, quercetin simultaneously activates Nrf2 to enhance antioxidant defences and inhibits Caspase-3 to attenuate apoptosis. Similarly, ginsenosides regulate mitochondrial homeostasis via the SIRT1/AMPK axis while inhibiting the p53/p21 pathway, thereby reducing senescent cell accumulation.

Clinically, modern drugs are mainly used to treat acute conditions or specific diseases, providing strong therapeutic effects but often accompanied by considerable side effects, such as the immunosuppressive effects of rapamycin. Conversely, natural small molecules are more suitable for chronic diseases and systemic regulation. For instance, resveratrol activates mitochondrial function through SIRT1, potentially delaying ageing; however, its long-term efficacy varies among individuals, posing challenges for clinical standardisation. With ongoing advances in computational biology and AI-driven compound screening, natural small molecules show great potential for integration into modern pharmacotherapy, particularly in the field of anti-ageing research.

## 6. Outlook

Ageing is an intricate biological process in which the same molecular factor may exert divergent effects under different microenvironmental contexts. Accordingly, this review primarily summarises the major functions of representative natural small molecules rather than all possible mechanisms. Future research on ageing should also take into account a broader range of ageing-related background factors, such as environmental exposure, lifestyle, and metabolic status, as these may influence both the interventional effects and the translational relevance of natural small molecules targeting oxidative stress and redox homeostasis. With ongoing advances in this field, future studies are expected to focus on mechanistic elucidation, optimisation of drug delivery strategies, exploration of combination therapies, and acceleration of clinical translation. These efforts will further promote the integration of natural small molecules into modern evidence-based medicine.

(1) Comprehensive elucidation of molecular targets and signalling pathways

Advances in single-cell sequencing, proteomics, metabolomics, and gene-editing technologies (e.g., CRISPR–Cas9) will provide more precise and in-depth insights into the molecular mechanisms of natural small molecules. For example, pathways such as FSP1/NADPH/CoQH_2_ and ferritinophagy play pivotal roles in ferroptosis regulation; however, investigations into the modulation of these pathways by natural small molecules remain limited. The integration of multi-omics approaches—including proteomics and metabolomics—will facilitate a more comprehensive understanding of the biological activities of natural small molecules and help uncover novel anti-ageing mechanisms.

(2) Optimisation of drug delivery systems

Natural small molecules often face challenges such as low solubility, rapid in vivo metabolism, and limited bioavailability, which constrain their clinical applicability. Future work should aim to enhance bioavailability and tissue-specific targeting through innovative drug delivery systems. For example, curcumin-loaded nanoliposomes have been shown to improve the solubility and stability of lipophilic compounds. In addition, targeted delivery systems may be developed to increase the specificity of natural small molecules for particular tissues or cell types. Novel formulations such as microemulsions and solid dispersions may further improve gastrointestinal absorption and reduce hepatic first-pass metabolism, thereby enhancing overall bioavailability.

(3) Exploration of combination therapies with modern pharmaceuticals

The concurrent use of natural small molecules and conventional drugs may exert synergistic therapeutic effects. For instance, co-administration of resveratrol with NAD^+^ precursors (e.g., nicotinamide mononucleotide, NMN) has been shown to delay cellular senescence, enhance mitochondrial function, and improve metabolic homeostasis. Further investigation into such combination regimens may yield more effective strategies for anti-ageing interventions.

(4) Promoting clinical translation of Natural Small Molecules

Despite substantial progress in experimental studies, only a few natural small molecules have advanced to clinical evaluation. Future studies should prioritise pharmacokinetic profiling, optimisation of dosing regimens, and preclinical safety assessments, followed by the initiation of Phase I–III clinical trials to promote their clinical implementation.

Internal and external environmental factors exert cumulative “wear and tear” on metabolic homeostasis, inflammatory responses, cellular repair, and extracellular matrix structure, thereby collectively driving the ageing process. Therefore, from the perspective of anti-ageing interventions, focusing solely on a single molecule or pathway is often insufficient. A more effective approach requires integrated regulation at multiple levels, including improving lifestyle, optimising metabolic status, reducing oxidative and inflammatory damage, and maintaining collagen integrity and tissue microenvironment homeostasis.

## 7. Conclusions

At the mechanistic level, individual Chinese herbal monomers delay ageing mainly through the following pathways: (1) activating antioxidant transcription axes such as Nrf2, FOXO, and SIRT1; (2) upregulating classical antioxidant enzymes, including SOD, CAT, and GPx; (3) inhibiting lipid peroxidation and ferroptosis; (4) promoting autophagy and mitophagy (via the Pink1/Parkin and BNIP3/NIX pathways) to maintain mitochondrial oxidative phosphorylation and membrane potential, thereby reducing mtDNA mutations and ROS leakage; (5) suppressing NF-κB and NLRP3 inflammasomes and decreasing SASP factors such as IL-6 and TNF-α to alleviate chronic inflammation; (6) modulating p16/p21/p53 and Bcl-2 family proteins to reverse cell cycle arrest and prevent excessive apoptosis; (7) mimicking caloric restriction (CR) by inhibiting the IGF-1/PI3K/AKT/mTOR axis while activating the AMPK/SIRT1/PGC-1α network, thus extending telomere length and facilitating DNA repair. Notably, the anti-ageing mechanisms of most natural small molecules are not confined to a single pathway but rather involve the coordinated regulation of multiple interconnected signalling networks, resulting in comprehensive protective effects.

Current evidence indicates that compounds such as ginsenosides, resveratrol, quercetin, curcumin, and cycloastragenol have shown anti-ageing-related effects in preclinical systems, including improvements in cognitive, musculoskeletal, photoageing, and metabolic phenotypes. Nevertheless, these findings are derived predominantly from cellular, nematode, *Drosophila*, and rodent models, whereas robust clinical evidence remains limited. Therefore, although these natural monomers represent promising candidates for further investigation, their translational relevance should be interpreted cautiously and requires confirmation through better-standardized preclinical studies and well-designed clinical trials.

External and internal environmental factors contribute to ageing through persistent “wear and tear” on metabolic homeostasis, inflammatory responses, cellular repair, and extracellular matrix integrity. Ultimately, these influences act together to drive the ageing process. Therefore, from the perspective of anti-ageing intervention, focusing solely on a single molecule or pathway is often insufficient. Instead, a more comprehensive strategy is needed, encompassing lifestyle improvement, optimisation of metabolic status, reduction in oxidative and inflammatory damage, and maintenance of collagen balance and tissue microenvironment homeostasis.

## Figures and Tables

**Table 1:** Mechanisms of Natural Small Molecules Regulating Antioxidant Aging Resistance via DAF-16.

Natural Small Molecules	Source	Experimental Model	Therapeutic Effect	Mechanism	References
Ginsenoside Rd	Ginseng	*Caenorhabditis elegans*	Enhanced the resistance of *C. elegans* to oxidative stress and related responses	Upregulated expression of antioxidant-related genes such as *sod-3*, *ctl-1*, *gst-4*	[21]
Crocin	*Crocus sativus* L.	*C. elegans* (wild type CL4176)	Significantly prolonged both mean and maximum lifespan of wild-type *C. elegans*	Activated DAF-16-related genes including *sod-3*, *gst-4*, *hsp-16.1*, *hsp-12.6*, thereby enhancing antioxidant enzyme activity (SOD, CAT)	[22]
Trigonelline	Fenugreek	*Caenorhabditis elegans*	Remarkably prolonged both mean and maximum lifespan of *C. elegans*	Activated DAF-16 and hsf-1 pathways; upregulated downstream antioxidant genes *sod-3*, *ctl-1*	[17]
Paeonol		*C. elegans*	Significantly increased both mean and maximum lifespan of *C. elegans*	Activated DAF-16/FOXO and SKN-1/Nrf2 signaling pathways	[23]
Nobiletin	Citrus fruits	*C. elegans*; Alzheimer’s disease model (CL4176, expressing Aβ)	Effectively delayed aging progression in *C. elegans* and extended lifespan	Upregulated *daf-16*, *sod-3*, *gcs-1* expression	[24]
Chrysophanol	Rhubarb	*C. elegans*	Significantly increased mean and maximum lifespan (extension by approximately 17–22%)	Acted through DAF-16 (FOXO) and SKN-1 (Nrf2 homolog) pathways, upregulating antioxidant genes *sod-3*, *gst-4*	[25]
Asperuloside	*Eucommia ulmoides* male flower	*Caenorhabditis elegans*, human skeletal muscle cells	Significantly extended the lifespan of *C. elegans*	Promoted nuclear translocation of DAF-16 and upregulated antioxidant and anti-aging genes	[26]
Naringenin	Citrus and grapefruits	*Caenorhabditis elegans*	Inhibited lipofuscin accumulation and prolonged lifespan	Upregulated *daf-16*, *sek-1*, and *skn-1* expression; activated *sod-3*, *ctl-1*, and *ctl-2*	[27]
Quercetin	*Sophora japonica* (Pagoda tree)	*Caenorhabditis elegans*	Significantly extended mean lifespan; reduced ROS and lipid accumulation	Activated DAF-16, thereby regulating *sod-1*, *sod-2*, *ctl-2*, and *gst-4*	[28]
Phloretin		*Caenorhabditis elegans*	Prolonged lifespan of *C. elegans* exposed to oxidative stress	Regulated FOXO/DAF-16 pathway, enhanced SOD and CAT activities	[29]
complanatoside A	Semen Astragali Complanati	*Caenorhabditis elegans*	Extend the lifespan of *Caenorhabditis elegans* and delay the progression of chronic diseases.	Required FOXO/DAF-16 and NRF2/SKN-1 pathways; increased SOD and CAT activities;	[30]

**Table 2:** Mechanisms by which Natural Small Molecules exert anti-aging effects via Nrf2-mediated antioxidant regulation.

Natural Small Molecules	Source	Model	Efficacy	Mechanism	References
Salvianolic acid B	*Salvia miltiorrhiza*	Human dermal fibroblasts exposed to UVB radiation	Protects fibroblasts against UVB-induced photoaging and oxidative damage.	Activates Nrf2 signaling pathway; upregulates SOD, CAT, and GSH-Px expression	[31]
Sulforaphane	*Cruciferous vegetables*.	Multiple models	Effectively prolongs cell lifespan and prevents neurodegeneration	Modulates Keap1/Nrf2 pathway; upregulates NQO1 and HO-1	[32]
Ginsenoside compound K	Ginseng	PC-12 aging cell model; aged mouse brain model	Delays brain aging	Enhances nuclear translocation of Nrf2; upregulates HO-1, SOD1, and GPX1 expression	[33]
Ginsenoside Rg1	Ginseng	LPS-induced cognitive impairment in mice	Improves learning and memory function in aging mice	Activates Nrf2 and downstream antioxidant enzymes HO-1 and NQO1	[34]
Hesperetin		Aged rat model	Improves oxidative stress in aged rats	Regulates Nrf2-Keap1 signaling; increases TrxR1 and SOD2 protein expression	[35]
Naringenin		D-galactose-induced pulmonary aging mouse model	Delay pulmonary aging	Activate the Nrf2/NQO1 pathway	[36]
Taxifolin (TAX)		D-galactose-induced aging mouse model	Delays aging process in D-galactose-induced aged mice	Regulates PI3K/AKT signaling, activates Nrf2, and increases the phosphorylation levels of HO-1 and NQO1	[37]
Nobiletin (Nob)		D-galactose-induced aging model of C2C12 myoblasts	Attenuates skeletal muscle aging and improves myotube atrophy	Upregulates Nrf2, reduces intracellular ROS levels	[38]
Berberine Hydrochloride	A variety of herbs	H_2_O_2_-induced oxidative stress model in C2C12 myoblasts	Reduces oxidative damage and protects C2C12 cells	Activates Nrf2/HO-1 signaling and enhances HO-1 activity	[39]

**Table 3:** Anti-ferroptosis and anti-aging effects of Natural Small Molecules.

Natural Small Molecules	Source	Model	Efficacy	Mechanism	References
Ginsenoside compound K (CK)	Ginseng	PC-12 senescent cell model; aged mouse model	Reduces brain aging and alleviates neurodegenerative disorders	Inhibits Nrf2 accumulation; suppresses ferroptosis-related enzymes GPX4 and SLC7A11 depletion; enhances iron storage, and reduces iron-mediated Fenton reaction	[33]
Ginsenoside Rg1	Ginseng	LPS-induced cognitive impairment in mice	Improves learning and memory deficits in aging mice	Activates Nrf2/SLC7A11/GPX4 signaling pathway	[34]
Rehmannioside A	*Rehmannia glutinosa* Libosch	Middle cerebral artery occlusion (MCAO) model in mice	Improves cognitive function	Activates SLC7A11/GPX4 pathway, promotes GSH synthesis and lipid peroxide elimination	[53]
Salidroside		Senescence-accelerated mouse model (SAMP8)	Alleviates cognitive decline and enhances energy metabolism	Activates Nrf2/GPX4 signaling; upregulates SLC7A11 and GPX4 protein expression	[54]
Thonningianin A		RSL-3-induced PC-12 cell model and Tau transgenic AD model	Significantly improves cognitive deficits in Alzheimer’s disease (AD)	Promotes GPX4 binding with ATF4, activates AMPK/Nrf2 signaling and upregulates GPX4, inhibits ferroptosis	[55]
Myricetin	*Myrica rubra*	Sevoflurane-induced cognitive impairment in aged mice	Alleviated cognitive impairment	Activates HDAC2/Nrf2/HO-1 signaling; upregulates GPX4 and SLC7A11 protein expression	[56]
Sennoside A	*Senna alexandrina*	APP/PS1 transgenic Alzheimer’s disease (AD) mouse model	Mitigates cognitive decline in AD mice	Reduced AD-induced iron (Fe^2+^) overload	[57]
Icariin		Rat model of osteoporotic fracture	Promotes osteoporotic fracture healing	Activates Nrf2 signaling; upregulates GPX4 and SLC7A11 expression	[58]
Quercetin		Natural aging chicken oocyte model	Reduces oocyte ferroptosis during aging	Upregulates SLC7A11, GPX4 and FTH1 expression, restoring iron homeostasis	[59]

**Table 4:** Antioxidant effects of Natural Small Molecules mediated by SIRT signaling.

Natural Small Molecules	Source	Model	Efficacy	Mechanism	References
Ginsenoside Rh4		D-galactose-induced model of skeletal muscle aging	Delays skeletal muscle senescence	Inhibits Nrf2 acetylation; upregulates SOD1/SOD2 and downregulates COX2 expression; activates SIRT1, regulates PGC-1α–TFAM and HIF-1α–c-Myc pathways to maintain mitochondrial homeostasis	[64]
Glycyrrhizin	Licorice roots	PMMA particle-induced aging mice	Attenuates bone loss and tissue aging in elderly mice	Promotes the expression of longevity-associated genes SIRT1 and SIRT6	[65]
Resveratrol		Aged zebrafish model	Alleviated the majority of age-related alterations in the retina	Activates the AMPK/Sirt1/PGC-1α pathway and promotes the expression of SOD and Gpx	[66]

**Table 5:** Anti-aging effects of Natural Small Molecules through activation of autophagy.

Natural Small Molecules	Source	Model	Efficacy	Mechanism	References
Asperuloside	*Eucommia ulmoides* male flower	*Caenorhabditis elegans*	Markedly attenuated muscle senescence	Activates mitochondrial autophagy	[26]
Icariin		H_2_O_2_-induced aging of BMSCs/macrophages	Attenuates age-related osteoporosis	Upregulates Atg and activates LC3	[76]
Hesperidin	Citrus species	*Caenorhabditis elegans*; AD models CL4176 and CL2006	Extend *C. elegans* lifespan	Upregulates acr-16 and autophagy-related genes (lgg-1, bec-1)	[77]
Resveratrol	Grape skin	Parkinson’s disease (PD) *Drosophila* model	Extend the lifespan of PD *Drosophila melanogaster*	Activates the PINK1/Parkin signaling pathway and increases the LC3-II/LC3-I ratio	[78]
Curcumin		IL-1β-induced chondrocyte model and rat osteoarthritis (OA) model	Delays cartilage degeneration in OA	Activates AMPK phosphorylation; promotes expression of PINK1, Parkin, LC3B, P62, and Beclin1 genes	[79]
Quercetin		Aging-related disease	Prevents aging-related disorders	Activates SIRT1/FOXO3-mediated autophagy and SIRT1–PINK1–Parkin mitophagy pathways	[80]
Oleanolic acid		MPP^+^-induced cellular and PD mouse models	Exerts neuroprotective effects against Parkinson’s disease	Regulates JNK–SPL–DJ-1 axis and upregulates mitochondrial protein DJ-1	[81]

**Table 6:** Effects of Natural Small Molecules on improving mitochondrial function.

Natural Small Molecules	Source	Model	Efficacy	Mechanism	References
Liquiritigenin		UV-induced photoaging cells	Reverses photoaging	Activates AMPK, promotes OXPHOS, and increases ATP production efficiency	[84]
Crocetin		Aged *C57BL/6J* mice	Delays brain aging	Regulates cellular ATP and NAD levels, restores OXPHOS, and improves mitochondrial membrane potential	[85]
Resveratrol		Post-ovulation aging oocytes	Delays oocyte aging	Reverses the decline of mitochondrial membrane potential	[86]
Theaflavin-3′-gallate		UVB-induced HaCaT cells	Anti-photoaging	Increases mitochondrial membrane potential	[87]
Fisetin		Post-fertilization aged oocytes	Delays postovulatory oocyte aging in mice	Upregulates Sirt1; increases mitochondrial transcription factors Tfam, and mitochondrial genes Co2 and Atp8 expression	[88]
Quercetin		D-galactose-induced cardiomyocytes	Ameliorates the senescent phenotype of cells	Enhances mitochondrial membrane potential and regulates fusion and fission processes	[89]
4,4′-dimethoxychalcone		Oocytes cultured in vitro with DMC	Protects oocytes from postovulatory aging	Restores mitochondrial membrane potential	[90]
Protocatechuic acid		UVB-induced photoaging cells	Reverses UVB-induced photoaging	Restores mitochondrial membrane potential	[91]

**Table 7:** Natural Small Molecules delay aging by reducing inflammation.

Natural Small Molecules	Source	Model	Efficacy	Mechanism	References
Ononin		IL-1β– -induced chondrocytes	Improves chondrocyte inflammation	Reduces the secretion of SASP factors such as IL-1β, MMP-13, and TNF-α, and inhibits the activation of MAPK and NF-κB signaling pathways	[113]
Oleanolic Acid		TBHP-induced human keratinocytes (HaCaT)	Exerts cytoprotective effects	Inhibits iNOS expression and reduces inflammatory responses	[114]
Resveratrol		An annual fish Nothobranchius guentheri	Reverses oocyte aging	Upregulates SIRT1 and NRF2; inhibits NF-κB inflammatory signaling; reduces pro-inflammatory cytokines (IL-1β, TNF-α, IL-8)	[115]
Hesperidin		Bleomycin-induced pulmonary fibrosis mouse	Ameliorates age-related pulmonary fibrosis	Inhibits IL6/STAT3 signaling pathway	[116]
Ginsenoside Rg1	Ginseng	Aged mice (brain aging model)	Ameliorates age-related cognitive impairment	Decreases IL-6, TNF-α, and other inflammatory cytokines; increases anti-inflammatory cytokines (IL-4, IL-10)	[117]
EGCG		UV-induced HaCaT cells and human epidermis	Delays photoaging, increases collagen and elastin content	Decreases IL-6, IL-8, MMP-1, and MMP-9 expression	[118]
(-)-Epicatechin		High-fat diet-fed mice	Increased markers related to mitochondrial biogenesis and improved metabolic endothelial ageing	Activated sirtuin 1 and restored mitochondrial function through an eNOS-dependent mechanism	[119]
Phillyrin	Forsythia	IL-1β-induced mouse chondrocytes	Alleviates the progression of age-related osteoarthritis (OA) and relieves inflammation in patient	Inhibits NF-κB pathway and reduces IL-1β, TNF-α, COX-2, IL-6, and iNOS levels	[120]
Quercetin		Aged mice with cognitive impairment t	Improves spatial learning and memory	Reduces IL-1β and IL-18 protein levels via the Sirtuin1/NLRP3 pathway	[121]
cyanidin-3-O-glucoside, C3G		PA-induced endothelial dysfunction in HUVECs	Alleviated endothelial dysfunction	Reversed the PI3K/Akt axis, inhibited TLR4/IκBα activation, and restored the Akt/eNOS pathway	[122]
Piceatannol		Aged mice	Ameliorate endothelial dysfunction	Inhibit arginase and improve eNOS/NO-related function	[123]

**Table 8:** Natural Small Molecules delay aging by alleviating chronic inflammation.

Natural Small Molecules	Source	Model	Efficacy	Mechanism	References
Resveratrol		An annual fish *Nothobranchius guentheri*	Ovary of short-lived fish	Acts via the SIRT1/NRF2 pathway to reduce NF-κB p65, IL-1β, TNF-α, and IL-8 pro-inflammatory factors	[115]
Quercetin		H_2_O_2_-treated preadipocytes and mature adipocytes	Reduces the burden of senescent cells	Upregulates SIRT1; inhibits NF-κB activation and decreases SASP levels	[124]
Hesperidin		Bleomycin-induced pulmonary fibrosis mouse model	Alleviates pulmonary fibrosis in aged mice	Downregulates IL-6/STAT3 expression	[116]
Ginsenoside Rb1	Ginseng	Intestinal crypt cells from aged mice	Delays intestinal aging	Increases protein expression of SIRT1, SIRT3, and SIRT6	[125]
Icariin		D-galactose (D-gal)-induced human lung fibroblasts	Significantly delays senescence of IMR-90 lung fibroblasts	Regulates the SIRT1/NF-κB signaling pathway	[126]
Urolithin A	Tannins	D-galactose-induced ageing mice	Improved cognitive function in ageing mice	By targeting TLR4 signalling, inhibited NF-κB activation and reduced IκBα phosphorylation as well as MAPK/PI3K activation	[127]
Resveratrol		AS mouse model	Restored intestinal mucosal barrier function and ameliorated ankylosing spondylitis (AS) in mice	Reduced pro-inflammatory factors such as IL-6 and inhibited the NLRP3 inflammasome and the TLR4/NF-κB pathway	[128]
Kaempferol		Dextran sulfate sodium (DSS)-induced colitis mouse model (UC)	Modulated the gut microbiota to alleviate UC	Reduced the levels of IL-1β, IL-6, and TNF-α, and inhibited the LPS–TLR4–NF-κB axis	[129]

**Table 9:** Natural Small Molecules delay aging by regulating cell cycle arrest.

Natural Small Molecules	Source	Model	Efficacy	Mechanism	References
Icariin		D-galactose (D-gal)-induced human lung fibroblasts	Significantly delays senescence in IMR-90 human lung fibroblasts	Downregulates p53 and p21 activation and decreases Cav-1 protein levels	[126]
Ginsenoside Rg1	Ginseng	D-galactose (D-gal)-induced premature ovarian insufficiency (POI) mouse model	Improves ovarian pathology	Decreases p21 and p53 expression levels	[134]
Fisetin		IL-1β-treated chondrocytes	Delays cartilage aging	Suppresses p16 and p21 expression, reducing cell cycle arrest	[135]
Quercetin		D-galactose (D-gal)-induced Wistar rats	Delays aging	Downregulates p53 and p21 expression levels	[136]
Hesperidin		Bleomycin-induced pulmonary fibrosis mouse model	Alleviates pulmonary fibrosis in aged mice	Downregulates p53, p21, and p16 expression levels	[116]
Curcumin		Senescent cells with γ-radiation-induced mitochondrial dysfunction; male Wistar albino rats	Improves radiation-induced neurotoxicity and delays cellular senescence	Suppresses p53, p21, and p16 mRNA expression levels	[137]
Resveratrol		Prematurely aged telomerase-deficient (terc^−^/^−^) mice	Attenuates age-related pulmonary dysfunction	Activates SIRT1, reduces BAX expression, and decreases p53 stability	[138]
EGCG		H_2_O_2_-induced adipocytes	Reduced the senescence burden across multiple tissues	Inhibited the PI3K/Akt/mTOR pathway and suppressed the accumulation of the anti-apoptotic protein Bcl-2	[139]

**Table 10:** Anti-aging effects of Natural Small Molecules under calorie restriction-mimetic conditions.

Natural Small Molecules	Source	Model	Efficacy	Mechanism	References
Hesperidin		Naturally aged mice	Improves age-related changes, restores a youthful metabolic profile	Upregulates *Cisd2* expression, mimics calorie restriction (CR), enhances mitochondrial homeostasis and function	[151]
Naringin	Citrus fruit	High-glucose-induced (HGI) *C. elegans*	Extends *C. elegans* lifespan	Depends on autophagy-related genes (*lgg-1*); reduces lipid accumulation	[152]
Quercetin		D-galactose (D-gal)-induced Wistar rats	Delays aging	Reduces renal GCLC and GCLM expression; increases pancreatic insulin and glucagon levels	[136]
Ginsenoside 20(*S*)-Rg3	Ginseng	Replicative senescence in human dermal fibroblasts (HDF)	Specifically reverses senescence in HDF cells	Activates *SIRT*/PGC1α signaling, improves mitochondrial metabolism	[153]
Resveratrol		D-galactose (D-gal)-treated rats	Exhibits calorie restriction (CR)-like anti-aging effects	Activates *SIRT1* and *FOXO3a*; upregulates mitochondrial activity	[154]
Cis-resveratrol		Aging-related neurodegeneration associated with serum tyrosine levels	Shows protective “CR mimetic”-like effects, potentially delaying aging and neurodegenerative diseases	Promotes eEF2 dephosphorylation to enhance the translation elongation process; simultaneously activates PARP1 auto-ADP-ribosylation and facilitates histone serine ADP-ribosylation, thereby enhancing DNA repair capacity	[155]
Oleanolic acid		Replicative senescence in human dermal fibroblasts (HDF)	Delays aging progression	Regulates IGF-1/PI3K/AKT/mTOR signaling, reduces IL-1β, IL-6, and IL-8 expression	[156]

## Data Availability

No new data were created or analyzed in this study.

## Abbreviations

The following abbreviations are used in this manuscript:

AMPK	AMP-Activated Protein Kinase
BNIP3	BCL2/adenovirus E1B 19 kDa-Interacting Protein 3
CR	Calorie Restriction
FOXO	forkhead box protein O
GPx	Glutathione Peroxidase
GPX4	Glutathione Peroxidase 4
HO-1	Heme Oxygenase-1
mtDNA	Mitochondrial DNA
MDA	Malondialdehyde
NLRP3	NLR family pyrin domain containing 3
Nrf2	Nuclear Factor Erythroid 2-Related Factor 2
NQO1	NAD(P)H Quinone Dehydrogenase 1
NF-κB	Nuclear Factor kappa-light-chain-enhancer of activated B cells
OXPHOS	Oxidative Phosphorylation
PGC-1α	Peroxisome Proliferator-Activated Receptor Gamma Coactivator 1 Alpha
PINK1	PTEN-Induced Kinase 1
Parkin	Parkin RBR E3 Ubiquitin Protein Ligase
ROS	Reactive Oxygen Species
SIRT1	Sirtuin 1
SASP	senescence-associated secretory phenotype
ΔΨm	Mitochondrial Membrane Potential
CDK–Cyclin	Cyclin-dependent kinase–Cyclin
BAX	Bcl-2-associated X protein
BCL-2	B-cell lymphoma 2
